# Frequency of Red Cell Alloimmunization and Autoimmunization in Thalassemia Patients: A Report from Eastern India

**DOI:** 10.1155/2015/610931

**Published:** 2015-09-06

**Authors:** Suvro Sankha Datta, Somnath Mukherjee, Biplabendu Talukder, Prasun Bhattacharya, Krishnendu Mukherjee

**Affiliations:** ^1^Department of Transfusion Medicine, The Mission Hospital, Durgapur, West Bengal 713212, India; ^2^Department of Transfusion Medicine, AIIMS, Bhubaneswar 751019, India; ^3^Department of Immunohematology & Blood Transfusion, MCH, Kolkata 700073, India

## Abstract

*Introduction*. Red blood cell (RBC) alloimmunization and autoimmunization remain a major problem in transfusion dependent thalassemic patients. There is a paucity of data on the incidence of RBC alloimmunization and autoimmunization in thalassemic patients from eastern part of India, as pretransfusion antibody screening is not routinely performed. *Aims*. To assess the incidence of RBC alloimmunization and autoimmunization in transfusion dependent thalassemic patients in eastern India. *Materials and Methods*. Total 500 thalassemia cases were evaluated. The antibody screening and identification were performed with commercially available panel cells (Diapanel, Bio-rad, Switzerland) by column agglutination method. To detect autoantibodies, autocontrol and direct antiglobulin tests were carried out using polyspecific coombs (IgG + C3d) gel cards in all patients. *Results*. A total of 28 patients developed RBC alloimmunization (5.6%) and 5 patients had autoantibodies (1%). Alloantibody against c had the highest incidence (28.57%) followed by E (21.42%). Five out of 28 (17.85%) patients had developed antibodies against both c and E. *Conclusion*. Data from this study demonstrate that the RBC alloantibody and autoantibody development rates are significant in our region. Thus, pretransfusion antibody screening needs to be initiated in eastern India in order to ensure safe transfusion practice.

## 1. Introduction

Thalassemia is a congenital hemolytic disorder, caused by a partial or complete defect in *α* or *β* globin chain synthesis. In India, it is estimated that around 8000–10000 new thalassemics (homozygous) are born every year and beta thalassemia gene is found more commonly in Punjabis, Sindhis, Bengalis, and Gujaratis [1]. In the absence of stem cell transplantation, the disease is treated by life-long red blood cell (RBC) transfusion [2] to keep the hemoglobin (Hb) level between 9 and 11.5 g/dL. Blood transfusion, despite being life-saving process, is associated with inherent risks of alloimmunization against red cells antigens. Red blood cell (RBC) alloimmunization occurs due to genetic disparity between donor and recipient red cells antigens [3]. The development of alloantibodies and autoantibodies against RBC antigens causes laboratory difficulties during RBC crossmatching, shortens in vivo survival of transfused red cells, delays provision of safe transfusions, and may accelerate iron overloading [4, 5]. Alloimmunization rates were reported from 4% to 50% in thalassemic patients and were lower in more homogenous populations [2, 6–8]. Red cells autoantibodies are not very common but they can result in clinical hemolysis and can cause difficulty during crossmatching. Patients with autoantibodies may have a higher transfusion rate and often require immunosuppressive drugs or splenectomy [9, 10]. The term “clinically significant” in relation to alloantibodies may refer to an antibody that causes an obvious, clinical hemolytic transfusion reaction (fever, chills, hemoglobinuria, etc.) or an antibody that does not cause any overt clinical symptoms but is associated with laboratory signs of hemolysis (increased bilirubin, decreased haptoglobin, etc.) or an antibody that is not associated with any clinical or laboratory signs of hemolysis, but RBCs incompatible with it survive less than normal lifespan [11]. In eastern part of India 5.6% of population have beta thalassemia trait and 5% of population have HbE carrier state [12]. But there is a paucity of data on the incidence of RBC alloimmunization and autoimmunization in thalassemic patients from this region, as pretransfusion antibody screening is not routinely performed. Thus this study was conducted to find out the frequency of alloimmunization, autoimmunization, and most common alloantibodies involved to red cell antigens in thalassemic patients. This study helped the authors to formulate transfusion strategies for all multitransfused thalassemic patients in eastern part of India.

## 2. Materials and Methods

The prospective and observational study was carried out in the Department of Immunohematology & Blood Transfusion, Medical College Hospital Kolkata, for the period of two and half years (January 2012 to June 2014). The study population were all transfusion dependent thalassemic patients of Medical College Hospital Kolkata. Informed consent was obtained from patients or their parents. The study was approved by hospital ethics committee.

### 2.1. Patients

Total 500 thalassemic patients were evaluated in the age ranging from 2 to 40 years. The inclusion criteria were patients who were dependent on transfusion and had a history of blood transfusion at least once in every month. The exclusion criteria were female patients who were transfusion dependent but had a history of Rh isoimmunization or fetomaternal haemorrhage. Clinical and transfusion records were analyzed in all patients for presence of alloimmunization/autoimmunization with antibody specificity among different age groups and different types of thalassemic (beta thalassemia major and E-beta thalassemia) patients.

### 2.2. Transfusion Policy

All thalassemia patients were transfused according to institutional transfusion policy to keep target Hb level 9–11.5 g/dL with a transfusion interval of 2–4 weeks (median interval of 3 weeks). As per transfusion strategy of our institute, all thalassemia patients were given ABO and Rh(D) matched packed red cells after compatibility testing by gel card technique in the AHG phase (type and crossmatch policy). In case patients were detected to have alloantibodies, those patients received ABO & Rh(D) matched particular antigen negative (against which they had alloantibody) compatible units for transfusion. Patients who had developed autoantibodies received transfusion with “best matched” units.

### 2.3. Immunohematological Tests

A volume of 2 mL blood was drawn into an ethylene diamine tetraacetate (EDTA) containing tube, centrifuged at 3000 ×g for 3 minutes to obtain plasma (for crossmatch and antibody screening) and red cells (for detection of autoantibodies) on gel card system. Prior to every transfusion, plasma was tested for the presence of alloantibodies by using commercial three-cell panel (Diacell, Bio-Rad, Switzerland). All alloantibody screening positive samples were evaluated to identify the antibody specificity. Antibody specificity detection was performed using a commercial 11-cell identification panel (Diapanel, Bio-Rad, Switzerland). Autocontrol was performed in each case to identify autoantibodies. It was done by incubating patient's cell with patient's plasma at 37°C for 15 minutes and then centrifuging for 10 minutes on gel card containing polyspecific antihuman globulin (anti-IgG + C3d). A polyspecific direct antiglobulin test was also performed each time using 1% cell suspension of the patient's RBC with antihuman globulin. All the tests were performed using the gel card method by Diamed ID (Switzerland), as per manufacturer's guidelines. Elution and adsorption methods were employed in patients with suspected autoantibodies.

### 2.4. Statistical Analysis

Statistical analysis was performed through SPSS software (version 17.0; SPSS Inc., Chicago, IL, USA) by making the frequency distribution tables and identifying frequency of alloimmunization and autoimmunization as well as the specificity of the particular alloantibodies. Discrete categorical data were presented as *n* (%). Comparisons for categorical data were made by Chi-square test. All reported *p* values are two-sided, with a significance level of 0.05.

## 3. Results

### 3.1. Patient Characteristics

**Figure 1:**
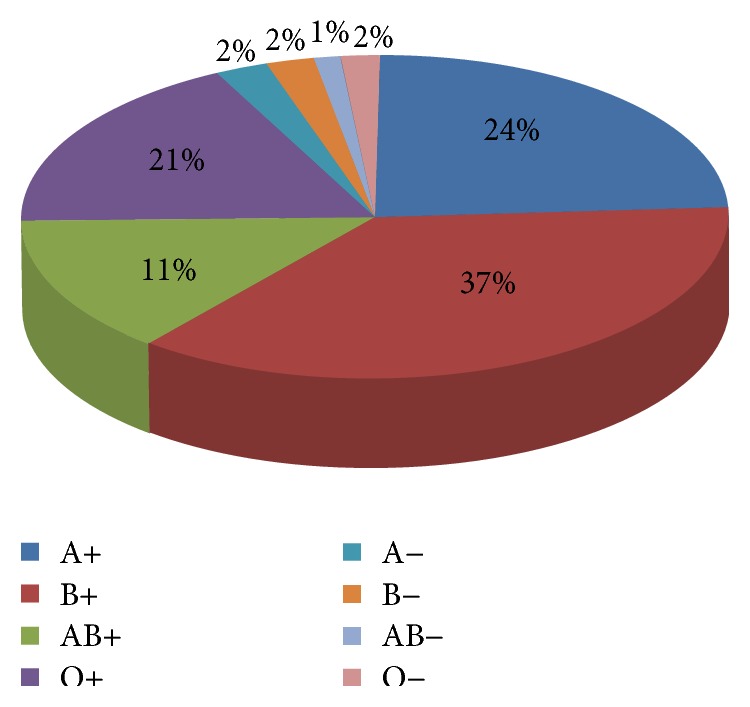
Blood groups of patients.

During the study period, a total of 500 thalassemia patients were reviewed. Three hundred thirty-three patients (66.6%) had beta thalassemia major and one hundred sixty-seven (33.4%) had E-beta thalassemia or thalassemia intermedia. There were 215 males and 285 females. Male to female ratio in this study was 1 : 1.33. According to blood group of the patients among Rh(D) positive patients, 120 of them were A, 184 of them were B, 106 of them were O, and 52 of them were AB. On the other side among Rh(D) negative patients, 12 of them were A, 11 of them were B, 9 of them were O, and 6 of them were AB (Figure 1).

### 3.2. Alloimmunization Rate in Different Groups according to Age, Sex, and Thalassemia Subtypes

**Figure 2:**
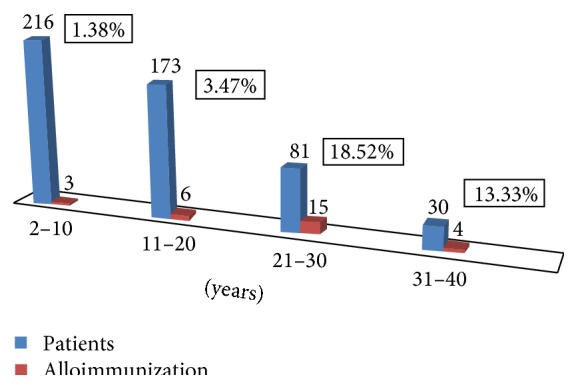
Alloimmunization in different age groups.

According to age of distribution patients were divided into 4 age groups. In between 2 and 10 years of age total patients were 216 and among them 3 cases of alloimmunization were detected (1.38%). In 11–20 years of age total 6 patients had alloantibodies among 173 patients (3.47%). In 21–30 years of age total patients were 81 and among them 15 had alloantibodies (18.52%). In 31–40 years of age among 30 patients 4 had alloimmunization (13.33%). So it was found that the rate of alloimmunization increases with the age of the patients' population and approximately 67.86% (19/28) of total cases of alloimmunization were detected in the age group of 21–40 years (Figure 2).

**Figure 3:**
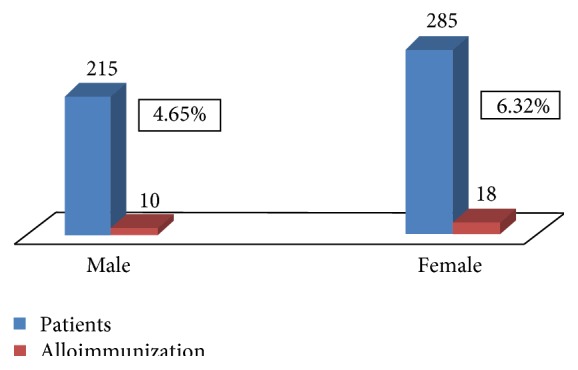
Alloimmunization in male and female patients.

The rates of alloimmunization among male and female patients population were 10 cases of alloimmunization among 215 of male patients and 18 cases of alloimmunization in 285 of female patients. The rates of alloimmunization among male and female populations were 4.65% (10/215) and 6.32% (18/285), respectively (Figure 3). As the *p* value was more than 0.05, there were no significant differences observed in rate of alloimmunization between male and female patients population.

The rate of alloimmunization was 5.71% (19/333) in beta thalassemia major patients and 5.39% (9/167) in E-beta thalassemic. As the *p* value was more than 0.05, therefore no significant differences were observed between beta thalassemia major and E-beta thalassemic patients (Table 1).

### 3.3. Alloantibody Specificity

**Figure 4:**
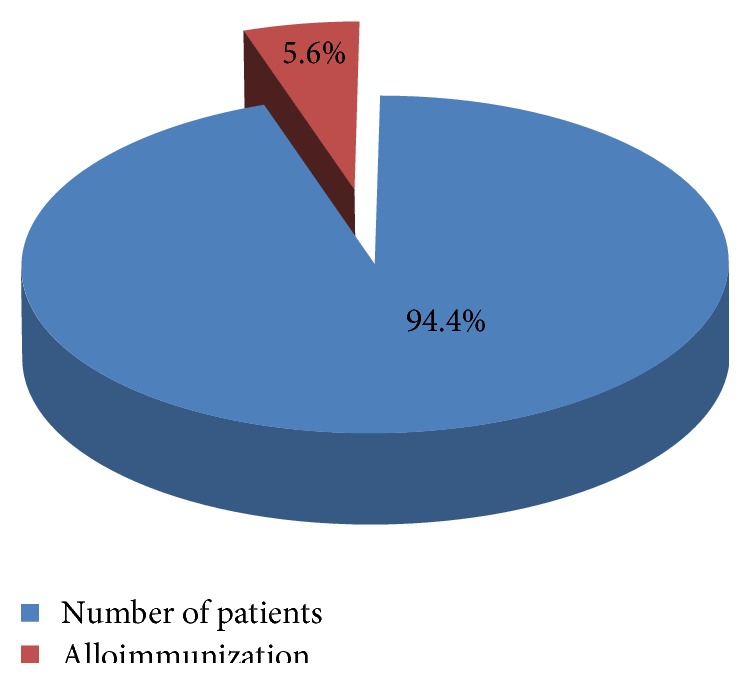
Alloimmunization rate.

A total of 28 patients developed RBC alloimmunization among 500 (5.6%) (Figure 4). Alloantibody against c had the highest incidence (28.57%) followed by E (21.42%), Jk^b^ (7.14%), Jk^a^ (3.57%), C (3.57%), D (3.57%), and s (3.57%), respectively. Five out of 28 (17.85%) patients had developed antibodies against c and E and three out of 28 patients had alloimmunization against C and D (3.57%), E and Jk^b^ (3.57%), and E and Fy^b^ (3.57%), respectively (Table 2).

### 3.4. Autoimmunization

Among 500 patients, 5 had (1%) developed autoantibodies as determined by positive autocontrol on gel card (IgG + C3d) as well as positive direct antiglobulin tests.

## 4. Discussion

There is no study on the frequency of alloimmunization and autoimmunization in transfusion dependent thalassemia patients from eastern part of India till now. In the present study we examined and defined the alloimmunization rate along with rate of autoantibody formation. We also reported the frequency of different alloantibodies in these patients' population that have not been previously described.

The factors for alloimmunization are complex and involve predominantly three contributing elements: the RBC antigenic difference between the blood donor and the recipient; the recipient's immune status; and the immunomodulatory effect of the allogeneic blood transfusions on the recipient's immune system. A low rate of alloimmunization may be expected when there is homogeneity of RBC antigens between the blood providers and recipients. Previous data on presumed homogenous populations in Greece and Italy showed an overall low rate (5% to 10%) of alloimmunization [6, 13, 14]. But data from Asia and Africa varies significantly in different countries. The rate of alloimmunization was reported as high as 23% in few countries [15, 16] to 7.7% in some other nations [17, 18] depending on demography and homogeneity of population. Different studies were reported from different parts of India showing the alloimmunization rate in between 3.4% and 8.6% [19–22]. In this study the rate of alloimmunization was 5.6% which was consistent with the rate of alloimmunization observed in other parts of India. At our center, most of the patients and blood donors are from West Bengal and adjoining areas. This homogeneity between the patient and blood donors population may be the reason for low rate of alloimmunization in this study.

When we compared the alloimmunization rate in different age groups, we found that most cases of alloimmunization (67.86%) were detected in the age group of 21–40 years as those patients were dependent on blood transfusion for several years. Thus it was assumed that the patients who required blood transfusion for several years with multiple units had more chance to form alloantibody in course of their life. This was consistent with a study which showed that frequency of alloantibody was higher among transfusion recipients of more than one unit of red cell transfusion and approximately 2–9% of those patients had new alloantibodies [23]. As we could not identify the actual starting time of blood transfusion in the study population, the low rate of alloimmunization in paediatric age group might be due to immune tolerance to form alloantibody on exposure to foreign red cell antigens [24, 25].

We did not find any association of gender (male/female) with rate of alloimmunization. In literature, few studies showed that gender was not a significant factor in the development of alloimmunization [26, 27]. However, some reported a significant association between alloimmunization and gender [28, 29]. Alloimmunization rate was not significantly affected depending on the diagnosis of thalassemia. It was almost same in beta thalassemia major and E-beta thalassemic patients.

In this study it was observed that around 78.5% of alloantibodies detected were against the antigens of Rh system. Similar result was also reported from a center in north India [19]. In this study alloantibody against c antigen was the most common alloantibody against a single red cell antigen (28.57%) followed by alloantibody against E (21.42%). Among the alloantibodies against multiple red cell antigens alloantibody against c and E was the most common (17.85%). This was consistent with the results of other studies [19, 21]. On obtaining a detailed transfusion history from the alloimmunized patients, it was found that the two Rh(D) negative patients who developed anti-D had received transfusions in rural hospitals on two and three occasions. We have no information about whether weak D testing of donor units was done at those hospitals. This could account for the development of anti-D in two of our thalassemic patients. It was reported that, in alloimmunized patients, the probability of additional antibody formation increases approximately threefold [30, 31]. This report alerts us that the transfusion dependent patients with single alloantibody are at risk of developing multiple alloantibodies in further course of time.

In the present study, 5 (1%) patients developed autoantibodies. Previously studies reported 1.7% to 11% rate of autoimmunization in thalassemia patients [32, 33]. No autoantibody was associated with alloimmunization in this study. In all cases elution was performed and elutes were tested with the panel cells on gel card. In all cases IgG autoantibodies were detected with a panagglutinin reaction with the panel cells. The clinical importance of autoantibodies in multitransfused patients is debatable. Although some reports found the existence of warm autoantibodies to be associated with clinically significant hemolysis [34], others did not find any significant association with hemolysis [35].

## 5. Conclusion

Although the overall incidence of RBC alloimmunization in this study was 5.6%, almost all of the alloimmunized patients had the antibodies which were clinically significant. Thus, pretransfusion antibody screening on patients' sample prior to crossmatching needs to be initiated in eastern India to ensure safe transfusion practice. We also recommended a practical, cost-effective, and feasible approach that the RBC antigen typing should be performed before first transfusion in thalassemic patients and issue of antigen matched blood (at least for Rh and Kell antigen) should be started to reduce the risk of alloimmunization.

## Figures and Tables

**Table 1:** Alloimmunization in different types of thalassemic patients.

Alloimmunization	Beta thalassemia major	E-beta thalassemia	*p* value
Rate	(19/333) = 5.71%	(9/167) = 5.39%	*p* > 0.05

**Table 2:** Alloantibody specificity.

Alloantibody type	Number of cases (*n*)	Frequency of alloantibody
Anti-c	8	(8/28) = 28.57%
Anti-E	6	(6/28) = 21.42%
Anti-(c + E)	5	(5/28) = 17.85%
Anti-Jk^b^	2	(2/28) = 7.14%
Anti-Jk^a^	1	(1/28) = 3.57%
Anti-(E + Fy^b^)	1	(1/28) = 3.57%
Anti-(E + Jk^b^)	1	(1/28) = 3.57%
Anti-C	1	(1/28) = 3.57%
Anti-D	1	(1/28) = 3.57%
Anti-(D + C)	1	(1/28) = 3.57%
Anti-s	1	(1/28) = 3.57%
Total (*n*)	28	
